# The Research and Evaluation of Antipsychotic Treatment in Community Behavioral Health Organizations, Outcomes (REACH-OUT) study: real-world clinical practice in schizophrenia

**DOI:** 10.1186/s12888-018-1594-1

**Published:** 2018-01-29

**Authors:** Kruti Joshi, Lian Mao, David M. Biondi, Robert Millet

**Affiliations:** 1 Janssen Scientific Affairs, LLC, 1125 Trenton-Harbourton Road, Titusville, NJ 08560 USA; 2 Janssen Research and Development, LLC, Titusville, NJ USA; 3 Carolina Behavioral Care, Durham, NC USA

**Keywords:** Antipsychotic therapy, Long-acting injectable, Community behavioral health organizations, Paliperidone palmitate, Risperidone long-acting injectable, Observational study, Schizophrenia, Patient outcomes, Real world

## Abstract

**Background:**

Outpatient facilities, such as community behavioral health organizations (CBHOs), play a critical role in the care of patients with serious mental illness, but there is a paucity of “real-world” patient outcomes data from this health care setting. Therefore, we conducted The Research and Evaluation of Antipsychotic Treatment in Community Behavioral Health Organizations, Outcomes (REACH-OUT) trial, a real-world, prospective, noninterventional observational study of patients with mental illness treated at CBHOs across the United States. We describe demographic and clinical characteristics, antipsychotic therapy (APT) treatment patterns, and health care resource utilization in patients with schizophrenia undergoing medical care as usual.

**Methods:**

This study enrolled adults with schizophrenia or bipolar I disorder who initiated APT treatment at various time points: 1) within 8 weeks of initiating risperidone long-acting injectables (RLAIs) or other APTs except paliperidone palmitate (PP), 2) after more than 24 weeks of continuous RLAI treatment, or 3) at any time after initiating PP LAI treatment (schizophrenia only). Study assessments were performed via participant interview, medical chart abstraction, and clinical survey at enrollment and at month 12.

**Results:**

A total of 1065 patients from 46 CBHOs were enrolled. Of these, 944 (88.6%) had a diagnosis of schizophrenia and 121 (11.4%) had bipolar I disorder. At enrollment, 599 (63.5%) of patients with schizophrenia were receiving RLAIs or PP LAI, 281 (29.8%) were receiving oral APTs, and 64 (6.8%) were receiving other injectable APTs. A number of differences in patient characteristics and outcomes were observed between patients in the LAI APT cohort and the oral APT cohort.

**Conclusion:**

Descriptive analyses from this observational study suggest differences in the patient characteristics, treatment patterns, and clinical and economic outcomes among those with schizophrenia treated at CBHOs with LAI APT or oral APTs. Additional analyses will be conducted to delineate the impact of LAI APT versus oral APTs on patient outcomes.

**Trial registration:**

Clinical Trial Registry: NCT01181960. Registered 12 August 2010.

**Electronic supplementary material:**

The online version of this article (10.1186/s12888-018-1594-1) contains supplementary material, which is available to authorized users.

## Background

For many patients with mental illness, community behavioral health organizations (CBHOs) are the primary source of care within the health care system. In the United States, the National Council for Behavioral Health coordinates nearly 2000 CBHOs, which serve approximately 6 million adults and children with mental illness and addiction disorders [1]. The fundamental goal of each CBHO is to improve the health and well-being of its community by delivering effective, accessible mental and behavioral health services. As the interface between primary care and mental health treatment, CBHOs play an essential role in the management and care of patients with mental illness [1, 2].

Schizophrenia is a serious mental illness that is complex and challenging to treat [1, 3]. Characterized by delusions, hallucinations, disorganized thinking and behavior, and social withdrawal [4], schizophrenia affects only about 1% of the US population [5], but it causes substantial financial burden, especially if not well controlled [6].

Since schizophrenia influences virtually all aspects of an individual’s life, it is important that clinicians and patients take a holistic approach when developing a treatment plan. Successful treatment must not only reduce or eliminate symptoms, but also maximize quality of life and social functioning and promote and maintain recovery. Treatment for schizophrenia typically requires a multidisciplinary approach that provides both psychopharmacology and psychosocial interventions [4]. Antipsychotic medications are the mainstay of treatment for patients with schizophrenia, but adherence to medication is often poor; this compromises outcomes and increases health care resource utilization [7]. Newer treatment innovations, such as atypical long-acting injectable (LAI) antipsychotic medications, have been effective in addressing poor adherence. By delivering therapeutic concentrations of medication continuously over the course of several days or weeks, LAI therapies eliminate the need for daily medication administration [8] and assure clinicians of patient adherence. Two such LAI antipsychotic therapies (APTs)—risperidone LAI (RLAI), an atypical APT administered once every 2 weeks for the maintenance treatment of patients with schizophrenia and bipolar I disorder [9], and paliperidone palmitate LAI (PP LAI), an atypical LAI APT administered once per month for the acute and maintenance treatment of patients with schizophrenia or schizoaffective disorder [10]—have demonstrated improved adherence and efficacy, as well as reduced relapse rates [11–14].

Historically, LAI APTs were reserved for patients with poor adherence and those who had failed other APTs (who typically have a longer history of disease) [15]. Although current clinical guidelines for patients with schizophrenia do not provide clear recommendations for LAI APT use, there is growing support for its use in first-episode psychosis, patients with frequent relapses, and those who prefer injectable over oral medication [11, 13, 15–21]. Despite the demonstrated therapeutic benefits of LAI APTs, they are prescribed at substantially lower rates in the United States (~8%) than elsewhere in the world (22%–36% in the United Kingdom, Belgium, Hong Kong, and Australia, for example) [17]. The reason for this discrepancy is unclear, and it underscores the need for a better understanding of how patients with schizophrenia in the United States are treated and managed.

Outpatient facilities, such as CBHOs, play a critical role in the care of patients with serious mental illness, but there is a paucity of patient outcomes data from this health care setting. This dearth of information is unfortunate given that prospective observational studies conducted in community settings provide valuable treatment usage and outcomes data. Unlike randomized controlled trials, which have stringent inclusion and exclusion criteria that limit external validity, prospective observational studies follow less restrictive methodological standards, and consequently their results are more generalizable to “real-world” practice settings [22]. To date, prospective observational studies evaluating the real-world treatment of patients with mental illness have been predominantly international studies, with limited data on current LAI APT usage and APT treatment outcomes in the United States [23–31]. Therefore, we conducted the Research and Evaluation of Antipsychotic Treatment in Community Behavioral Health Organizations, Outcomes (REACH-OUT) trial, a real-world observational study of patients with mental illness treated at CBHOs across the United States. The main objectives of REACH-OUT were to describe the demographic and clinical characteristics, APT treatment patterns, and health care resource utilization of patients with schizophrenia undergoing medical care as usual. By describing the characteristics of patients receiving APT in CBHOs, REACH-OUT will generate real-world data from naturalistic outpatient settings and provide health care providers, researchers, policy makers, and other stakeholders a holistic picture of schizophrenia treatment practices in the community setting. These data could then be used to evaluate associated outcomes that support clinical decision-making, best practices, and treatment guidelines. This article describes the study design of REACH-OUT and the patient characteristics and outcomes among LAI APT and oral APT cohorts at enrollment and after 12 months of follow-up.

## Methods

### Study design

The REACH-OUT study was a prospective, noninterventional, observational study of adult patients receiving their usual courses of treatment for schizophrenia or bipolar I disorder in CBHO settings in the United States (ClinicalTrials.gov number NCT01181960; Clinical Registry number CR017107). This Janssen Pharmaceuticals–sponsored study was conducted between August 2010 and November 2013, approved by participating ethics committees (Additional file 1: Table S1) and institutional review boards (New England Independent Review Board, Needham, MA), and conducted in accordance with the ethical principles of the Declaration of Helsinki. Written informed consent was obtained from all subjects prior to study enrollment. No intervention was provided in this study and APTs were not randomly assigned; all participants continued their usual course of treatment during the study period. This article describes the sociodemographic characteristics, psychiatric history, and clinical and economic outcomes of patients with schizophrenia treated with atypical LAI APTs and oral APTs.

### Study population

Study participants were recruited from 46 CBHOs in the United States that served as the primary sites of outpatient treatment. Adults aged ≥18 years who were diagnosed according to DSM-IV criteria with schizophrenia (disorganized, catatonic, paranoid, residual, or undifferentiated type) or bipolar I disorder (single manic episode or most recent manic, depressed, mixed, or unspecified episode) were eligible to participate. Patients with schizophrenia or bipolar I disorder were eligible to enter the study within 8 weeks of initiation or switch to RLAI or other APTs, or after >24 weeks of continuous RLAI treatment with no gaps between injections of more than 30 days. Patients with schizophrenia were eligible to enter the study at any time after clinician-ordered initiation of PP LAI in the 8 weeks prior to or on the day of enrollment (includes patients not previously on any APTs and those switched from another antipsychotic); or on continuous PP LAI for any time period prior to enrollment. Selection of the antipsychotic medication was at the discretion of the treating physician. Patients prescribed quetiapine at doses ≤200 mg/day for sleep were not eligible.

Eligible patients were grouped according to the APT that they received, resulting in an LAI APT cohort and an oral APT cohort. All patients prescribed oral antipsychotic medications were placed in the oral APT cohort, and the type of oral APT was not recorded. Patients were further categorized as either “new users” or “continuous users.” New users were defined as patients initiating their first or a different APT within 8 weeks (≤56 days) of enrollment. This included patients not previously taking any APT treatment and those switched from one APT to another. All patients receiving oral APT were considered new users. Continuous users were defined as patients taking LAI APT for more than 8 weeks (>56 days) prior to enrollment. The patients were followed prospectively for 12 months, during which time the participants received their medication per usual medical care in their usual treatment setting.

### Data collection

**Fig. 1:**
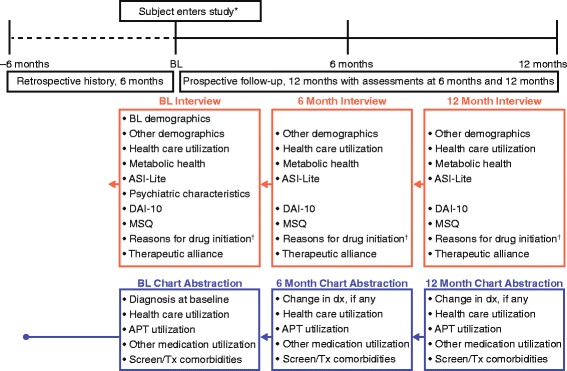
Study design. APT, antipsychotic; ASI-Lite, Addiction Severity Index Lite; BL, baseline (enrollment); DAI-10, Drug Attitude Inventory 10-item scale; Dx, diagnosis; LAI, long-acting injectable (includes paliperidone palmitate long-acting injectable and risperidone long-acting injectable); MSQ, Medication Satisfaction Questionnaire; PP LAI, paliperidone palmitate long-acting injectable; RLAI, risperidone long-acting injectable; Tx, treatment. *Entered within 8 weeks of start or switch to RLAI or other antipsychotic, after >24 weeks of continuous RLAI treatment, or any time after initiation of PP LAI. ^†^Asked of cohort that included participants newly initiated on an antipsychotic at baseline

Patient-reported outcomes were collected from the participants via face-to-face interviews conducted at enrollment and at 6- and 12-month follow-up visits (Fig. 1). At each visit, medical history and health care resource utilization information were abstracted from the participant’s medical charts (Fig. 1). The baseline chart abstraction (conducted at enrollment) collected 6-month retrospective data, whereas the 6- and 12-month chart abstractions collected on-study data covering the prior 6 months. The chart abstractions included data on diagnosis, psychiatric history, comorbidities, health care resource utilization, and APT utilization.

### Outcomes

Patient demographic and clinical characteristics, comorbidities, and psychiatric history were collected at enrollment, along with prior health care resource utilization and patient-reported outcomes. The key outcomes assessed were APT utilization, health care resource utilization (including inpatient hospitalization, emergency department visits, and outpatient services), patient-reported outcomes (ie, attitude toward medication, medication satisfaction, quality of life, and social engagement and patient–caregiver engagement), patient functioning, symptom remission, reasons for initiation of medication, and suicidality.

Patients’ attitudes about medication were assessed using the Drug Attitude Inventory 10-item scale (DAI-10) [32]. Patient satisfaction with medication was assessed with the Medication Satisfaction Questionnaire (MSQ), a one-item satisfaction question that is measured using a 7-point Likert scale, with response options ranging from “1 – very dissatisfied” to “7 – very satisfied.” A change of 1 point in MSQ is considered clinically meaningful [33]. Patients’ quality of life was assessed using a modified version of Lehman’s Quality of Life Interview [34], and alcohol and substance abuse were measured with the Addiction Severity Index–Lite (ASI-Lite) [35]. Patient engagement and interactions with clinicians, family, friends, and support networks during the past 4 weeks were assessed with six questions asked during the face-to-face interviews. Answers to these questions were used to determine whether or not patients were more or less engaged with family, friends, and others. Patient functioning was measured using the Personal and Social Performance (PSP) scale [36]. Higher PSP scores indicate better functioning. Remission was measured using the Structured Clinical Interview for Symptoms of Remission (SCI-SR) [37, 38]. The InterSePT Scale for Suicidal Thinking–Plus (ISST-Plus) short form [39] was used by clinicians to assess patients’ suicide ideation and behavior. Clinician participants were queried as to why patients who initiated a new antipsychotic at baseline were initiated on that medication (LAI or oral antipsychotic, as applicable). The reasons for initiation will include insufficient response, patient choice, compliance issues, adverse events, tolerability, or other reasons.

### Statistical methods

Sample sizes needed to achieve various precisions (95% CI) of estimation for an event rate were calculated. To achieve a precision of ±3%, it was predetermined that approximately 1068 patients were needed (when the true event rate was 50%).

Given the observational nature of this study, the primary method of analysis was descriptive. The primary patient cohorts of interest were the LAI APT cohort and oral APT cohort. The LAI APT cohort consisted of all patients with schizophrenia who were PP LAI or RLAI users at the time of enrollment. The oral APT cohort consisted of all patients with schizophrenia who were oral APT users at enrollment.

Descriptive summaries included means and standard deviations (SD) for continuous measures and counts and percentages for categorical measures. To assess cohort differences at study enrollment, a chi-squared test was used to compare categorical variables, and a two-sample t-test was used to compare continuous variables. The objective of these comparative analyses was to investigate whether patient cohorts at enrollment were comparable. No adjustments were made for multiplicity.

## Results

### Patient disposition

A total of 1065 patients were enrolled from 46 CBHOs in the United States. Of these, 944 (88.6%) had a diagnosis of schizophrenia (Table 1) and 121 (11.4%) had a diagnosis of bipolar I disorder. A total of 682 (72.2%) of the 944 patients with schizophrenia completed the 12-month follow-up study period, whereas 103 (85.1%) of the 121 patients with bipolar I disorder completed the 12-month study follow-up. This article describes the characteristics and outcomes of the patients with schizophrenia at enrollment and at the 12-month follow-up visit.

**Table 1:** Overview of the schizophrenia patient population

	LAI New User	LAI Continuous User	LAI Total	Oral APT	Other Injectable/Unknown	Study Total
	*n* = 235	*n* = 385	*n* = 620	*n* = 377	*n* = 68	*N* = 1065
Schizophrenia						
Baseline	214	385	599	281	64	944
Month 6	173 (80.8)	326 (84.7)	499 (83.3)	230 (81.9)	40 (62.5)	769 (81.5)
Month 12	156 (72.9)	290 (75.3)	446 (74.5)	204 (72.6)	32 (50.0)	682 (72.2)

*APT* antipsychotic therapy, *LAI* long-acting injectable, *NA* not applicable

Among the 599 patients with schizophrenia in the total LAI group, 482 were receiving PP LAI (174 new users; 308 continuous users) and 117 were receiving RLAI (40 new users; 77 continuous users)

Values in parentheses are percentages unless otherwise specified

*New user* was defined as a study patient who initiated PP LAI or RLAI treatment within 8 weeks (≤56 days) of the enrollment period

*Continuous user* was defined as a study patient who initiated PP LAI or RLAI treatment more than 8 weeks (>56 days) after the enrollment visit

### Patient characteristics and comorbidities

**Fig. 2:**
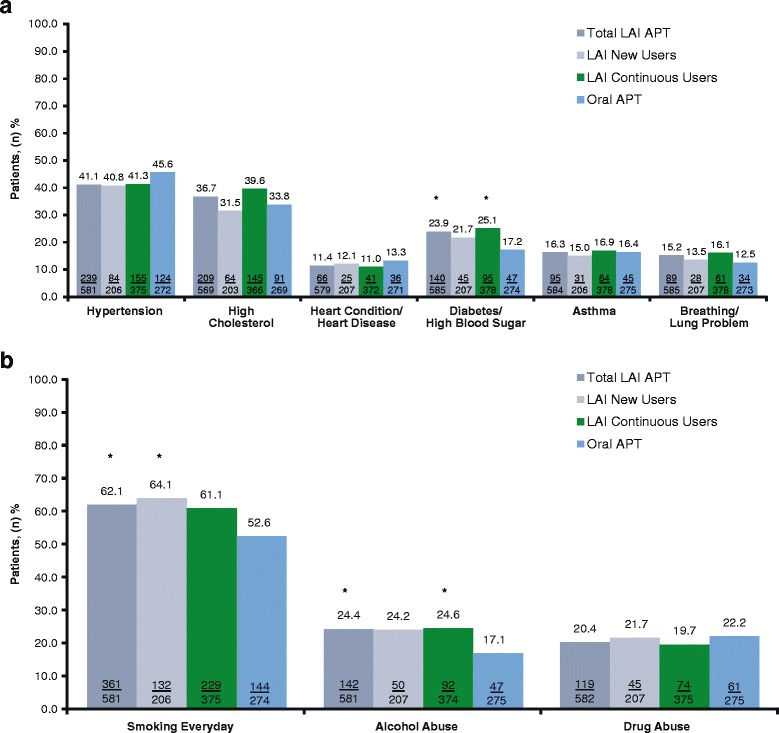
Comorbid conditions at enrollment (all patients with schizophrenia). Medical (**a**) and behavioral (**b**) comorbid conditions. APT, antipsychotic therapy; LAI, long-acting injectable (includes paliperidone palmitate long-acting injectable and risperidone long-acting injectable). Values in bars are n/N. *P < 0.05 vs oral APT

The patients with schizophrenia were predominantly men (72.5%, total LAI APT cohort; 65.8%, oral APT cohort) who were mostly white (50.6%, total LAI APT cohort; 49.1%, oral APT cohort) or black (32.8%, total LAI APT cohort; 33.2%, oral APT cohort), and were ~41 years of age (range, 18–80 years) (Table 2). Demographic and socioeconomic characteristics were generally similar among treatment groups, except patients in the total LAI APT cohort were less likely to be Spanish/Hispanic/Latino, have higher levels of education, reside in a supervised group living situation, be single (never married), and receive Medicare or Medicaid than those in the oral APT cohort (Table 2). Patients in the total LAI APT cohort were also more likely to have a longer disease history (Table 3). Patients in the LAI APT cohorts were more likely to be smokers, diabetic, and alcohol abusers compared with those in the oral APT cohort (Fig. 2).

**Table 2:** Sociodemographic characteristics at enrollment of long-acting injectable antipsychotic therapy vs oral antipsychotic therapy users^a^

	LAI APT			
	Total *n* = 599	New User *n* = 214	Continuous User *n* = 385	Oral APT *n* = 281
Age, years				
N	566	200	366	265
Mean (SD)	41.1 (12.42)	39.4 (12.01)	42.0 (12.56)	42.1 (13.49)
*P* value (LAI vs oral)	0.268	0.021	0.921	
Sex, *n* (%)				
N	585	207	378	275
Male	424 (72.5)	155 (74.9)	269 (71.2)	181 (65.8)
Female	161 (27.5)	52 (25.1)	109 (28.8)	94 (34.2)
*P* value (LAI vs oral)	0.054	0.031	0.144	
Race, *n* (%)				
N	577	203	374	271
White	292 (50.6)	85 (41.9)	207 (55.3)	133 (49.1)
Black or African American	188 (32.6)	90 (44.3)	98 (26.2)	90 (33.2)
Asian, Native Hawaiian, or other Pacific Islander	9 (1.6)	3 (1.5)	6 (1.6)	1 (0.4)
American Indian or Alaska Native	12 (2.1)	3 (1.5)	9 (2.4)	8 (3.0)
Multiracial/Other	76 (13.2)	22 (10.8)	54 (14.4)	39 (14.4)
*P* value (LAI vs oral)	0.645	0.092	0.276	
Ethnicity (Spanish, Hispanic, Latino), *n* (%)				
N	576	200	376	274
No	494 (85.8)	174 (87.0)	320 (85.1)	202 (73.7)
Yes	82 (14.2)	26 (13.0)	56 (14.9)	72 (26.3)
*P* value (LAI vs oral)	<0.001	<0.001	<0.001	
Education, *n* (%)				
N	584	207	377	272
8th grade or less	56 (9.6)	25 (12.1)	31 (8.2)	45 (16.5)
Some high school, did not graduate/get GED	161 (27.6)	56 (27.1)	105 (27.9)	70 (25.7)
High school degree/GED	222 (38.0)	70 (33.8)	152 (40.3)	101 (37.1)
Some college or college degree	145 (24.8)	56 (27.1)	89 (23.6)	56 (20.6)
*P* value (LAI vs oral)	0.030	0.234	0.019	
Military service, *n* (%)				
N	581	206	375	273
No	535 (92.1)	187 (90.8)	348 (92.8)	255 (93.4)
Yes	46 (7.9)	19 (9.2)	27 (7.2)	18 (6.6)
*P* value (LAI vs oral)	0.577	0.309	0.868	
Marital status, *n* (%)				
N	584	206	378	275
Single, never married	413 (70.7)	149 (72.3)	264 (69.8)	160 (58.2)
Married	37 (6.3)	9 (4.4)	28 (7.4)	27 (9.8)
Widowed	13 (2.2)	2 (1.0)	11 (2.9)	8 (2.9)
Divorced/Separated	108 (18.5)	41 (19.9)	67 (17.7)	72 (26.2)
Nonmarried committed relationship	13 (2.2)	5 (2.4)	8 (2.1)	8 (2.9)
*P* value (LAI vs oral)	0.010	0.012	0.035	
Living situation, n (%)				
N	586	207	379	273
Clinical facility^b^	10 (1.71)	6 (2.90)	4 (1.06)	2 (0.73)
Supervised group living (generally long-term)	67 (11.4)	15 (7.2)	52 (13.7)	7 (2.6)
Transitional group home (halfway/quarterway house)	3 (0.5)	1 (0.5)	2 (0.5)	3 (1.1)
Family foster care	1 (0.2)	0 (0.0)	1 (0.3)	1 (0.4)
Cooperative apartment	20 (3.4)	8 (3.9)	12 (3.2)	9 (3.3)
Boarding home	47 (8.0)	27 (13.0)	20 (5.3)	17 (6.2)
Private house or apartment	410 (70.0)	139 (67.1)	271 (71.5)	212 (77.7)
Other	28 (4.8)	11 (5.3)	17 (4.5)	22 (8.1)
*P* value (LAI vs oral)	0.001	0.036	<0.001	
Medicare, n (%)				
N	575	202	373	266
No	275 (47.8)	106 (52.5)	169 (45.3)	166 (62.4)
Yes	300 (52.2)	96 (47.5)	204 (54.7)	100 (37.6)
*P* value (LAI vs oral)	<0.001	0.036	<0.001	
Medicaid or medical assistance, *n* (%)				
N	574	202	372	270
No	140 (24.4)	61 (30.2)	79 (21.2)	101 (37.4)
Yes	434 (75.6)	141 (69.8)	293 (78.8)	169 (62.6)
*P* value (LAI vs oral)	<0.001	0.115	<0.001	
Private health insurance, *n* (%)				
N	573	200	373	273
No	535 (93.4)	183 (91.5)	352 (94.4)	248 (90.8)
Yes	38 (6.6)	17 (8.5)	21 (5.6)	25 (9.2)
*P* value (LAI vs oral)	0.204	0.872	0.088	
Veterans or military medical benefits, *n* (%)				
N	580	203	377	273
No	569 (98.1)	200 (98.5)	369 (97.9)	271 (99.3)
Yes	11 (1.9)	3 (1.5)	8 (2.1)	2 (0.7)
*P* value (LAI vs oral)	0.242	0.646	0.202	

*APT* antipsychotic therapy, *GED* general education diploma, *LAI* long-acting injectable (includes paliperidone palmitate long-acting injectable and risperidone long-acting injectable), *SD* standard deviation

^a^All subjects with schizophrenia. ^b^Includes hospital, skilled nursing facility (24-h nursing), and intermediate care facility (<24-h nursing)

**Table 3:** Psychiatric comorbidities at enrollment for long-acting injectable antipsychotic therapy vs oral antipsychotic therapy cohorts^a^

	LAI APT			
	Total *n* = 599	New Users *n* = 214	Continuous Users *n* = 385	Oral APT *n* = 281
Age when first hospitalized for symptoms of diagnosis, years				
*n*	480	164	316	180
Mean (SD)	24.2 (8.35)	24.1 (8.10)	24.3 (8.49)	26.2 (10.24)
*P* value (LAI APT vs oral APT)	0.020	0.034	0.033	
Time from first hospitalization to enrollment, years				
*n*	471	160	311	173
Mean (SD)	16.9 (12.11)	14.8 (11.94)	17.9 (12.08)	14.5 (11.39)
*P* value (LAI APT vs oral APT)	0.025	0.813	0.002	
Age when first diagnosed with schizophrenia or bipolar I, years				
*n*	287	91	196	105
Mean (SD)	25.9 (9.66)	25.3 (9.38)	26.1 (9.80)	29.1 (13.34)
*P* value (LAI APT vs oral APT)	0.022	0.020	0.042	
Time from diagnosis to enrollment, years				
*n*	275	86	189	102
Mean (SD)	15.0 (12.63)	12.2 (11.37)	16.3 (13.00)	11.9 (13.47)
*P* value (LAI APT vs oral APT)	0.041	0.890	0.008	
Age at first experience of symptoms, years				
*n*	475	164	311	196
Mean (SD)	22.3 (8.25)	21.9 (8.02)	22.5 (8.38)	23.9 (10.17)
*P* value (LAI APT vs oral APT)	0.048	0.033	0.107	
Time from first experience of symptoms to enrollment, years				
*n*	464	159	305	190
Mean (SD)	18.4 (12.26)	17.0 (12.14)	19.1 (12.28)	17.2 (12.41)
*P* value (LAI APT vs oral APT)	0.269	0.875	0.098	
Any psychiatric hospitalization prior to enrollment				
*n*	533	184	349	250
*n* (%)	118 (22.1)	59 (32.1)	59 (16.9)	55 (22.0)
*P* value (LAI APT vs oral APT)	1.000	0.018	0.140	
Time from the most recent psychiatric hospitalization to enrollment, days				
*n*	118	59	59	54
Mean (SD)	97.7 (139.32)	65.7 (48.13)	129.7 (186.42)	61.8 (43.91)
*P* value (LAI APT vs oral APT)	0.012	0.658	0.008	
Any all-cause hospitalization prior to enrollment				
*n*	533	184	349	250
*n* (%)	131 (24.6)	68 (37.0)	63 (18.1)	65 (26.0)
*P* value (LAI APT vs oral APT)	0.718	0.012	0.019	
Time from the most recent all-cause hospitalization to enrollment, days				
*n*	131	68	63	63
Mean (SD)	96.1 (133.62)	67.4 (50.56)	127.0 (181.11)	65.5 (45.95)
*P* value (LAI APT vs oral APT)	0.020	0.828	0.011	
Any psychiatric ED visit prior to enrollment				
*n*	495	166	329	229
*n* (%)	62 (12.5)	31 (18.7)	31 (9.4)	34 (14.8)
*P* value (LAI APT vs oral APT)	0.410	0.343	0.058	
Any all-cause ED visit prior to enrollment				
*n*	495	166	329	229
*n* (%)	85 (17.2)	41 (24.7)	44 (13.4)	41 (17.9)
*P* value (LAI APT vs oral APT)	0.830	0.104	0.153	

*APT* antipsychotic therapy, *ED* emergency department, *LAI* long-acting injectable (includes paliperidone palmitate long-acting injectable and risperidone long-acting injectable), *SD* standard deviation

^a^All subjects with schizophrenia

At study enrollment, approximately two-thirds (63.5%) of patients were in the total LAI APT cohort and approximately one-third (29.8%) were in the oral APT cohort. Of the 599 patients in the total LAI APT cohort, 482 (80.5%) and 117 (19.5%) were receiving PP LAI and RLAI, respectively, and 214 (35.7%) and 385 (64.3%) were new and continuous users of LAI APT. Among new and continuous users of PP LAI, 234 mg was the most common initial dose (59.0% vs 56.1%), followed by 156 mg (24.8% vs 27.4%) and 117 mg (14.3% vs 15.5%), respectively. For those prescribed RLAI, the most common starting dose among new users was 25 mg (48.5%), followed by 50 mg (27.3%) and 37.5 mg (21.2%); the most common starting dose among continuous users was 50 mg (71.1%), followed by 37.5 mg (14.5%) and 25 mg (7.9%).

At study enrollment, results assessed by ASI-Lite showed no significant difference between total LAI APT and oral APT cohorts in the mean (SD) number of days that the patient used alcohol in the past 30 days (1.8 [5.02] vs 1.7 [4.80] days; *P* = 0.933). However, there was a significant difference in nonalcohol substance abuse between total LAI APT and oral APT cohorts at enrollment: the mean (SD) number of days patients used sedatives, hypnotics, or tranquilizers in the past 30 days was significantly lower in patients in the total LAI APT cohort compared with those in the oral APT cohort (1.9 [7.14] vs 3.7 [9.74] days; *P* = 0.008). A similar trend was observed for new and continuous users of LAI APT (1.4 [6.03] and 2.2 [7.68] days, respectively; both *P* < 0.05 vs oral APT). The use of opiates or analgesics in the past 30 days was significantly lower in the total LAI APT cohort compared with the oral APT cohort (mean [SD] number of days: 0.7 [4.15] vs 1.9 [6.67] days; *P* = 0.009). Continuous users of LAI APT had fewer days using opiates or analgesics in the past 30 days compared with new users of LAI APT (0.6 [3.82] vs 0.9 [4.70]).

### Health care resource utilization

#### Hospitalizations and emergency department/crisis center

**Fig. 3:**
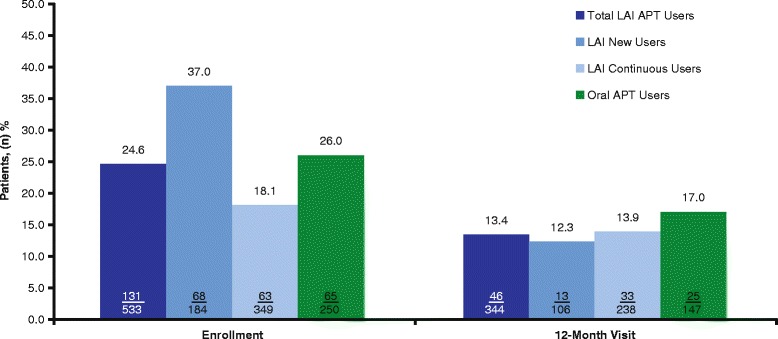
Proportion of patients hospitalized by time point (all patients with schizophrenia). APT, antipsychotic therapy; LAI, long-acting injectable (includes paliperidone palmitate long-acting injectable and risperidone long-acting injectable). Values in bars are n/N. Hospitalization at enrollment was defined as patients hospitalized in the 6-month pre-enrollment period. Total LAI APT = LAI new users + LAI continuous users

In the total LAI APT cohort, the proportion of patients who were hospitalized was 24.6% at the enrollment visit and 13.4% at the 12-month follow-up visit. A similar trend was observed among patients in the oral APT cohort; the proportion of patients who were hospitalized was 26.0% at enrollment and 17.0% at the 12-month follow-up visit (Fig. 3). At enrollment, twice as many new users of LAI APT had been hospitalized than continuous users of LAI APT (37.0% vs 18.1%, respectively), but this difference was not observed at the 12-month follow-up visit (12.3% and 13.9%, respectively). The proportion of patients who utilized emergency departments or crisis centers was 17.2% at the enrollment visit and 13.1% at the 12-month follow-up visit among those in the total LAI APT cohort, and 17.9% and 13.6% among those in the oral APT cohort. Utilization of emergency departments or crisis centers was higher in new users of LAI APT than continuous users of LAI APT at enrollment (24.7% vs 13.4%, respectively), but this difference was not observed at the 12-month follow-up visit (10.0% vs 14.3%, respectively).

#### Outpatient services

Patients in the total LAI APT cohort had utilized outpatient services more than patients in the oral APT cohort. At enrollment and at the 12-month follow-up visit, patients in the total LAI APT cohort were more likely to visit nurse practitioners, therapists/counselors, and nurses; attend group sessions; spend a greater number of days accessing day services at the CBHO; and sleep at a residential facility that was part of the CBHO than those in the oral APT cohort (Additional file 2: Table S2).

### Patient-reported outcomes

#### Attitude toward medication

Mean (SD) total DAI-10 scores were 6.0 (3.66) in patients in the LAI APT cohorts and 4.5 (4.03) in the oral APT cohort at the enrollment visit (Table 4). At the 12-month follow-up visit, mean (SD) total DAI-10 scores were 6.1 (3.83), 6.0 (3.68), and 6.1 (3.89) for total, new, and continuous users of LAI APT and 5.2 (4.10) for those in the oral APT cohort.

**Table 4:** Patient-reported outcomes at enrollment

		LAI APT			
Patient-Reported Outcome	Assessment	Total *n* = 599	New Users *n* = 214	Continuous Users *n* = 385	Oral APT *n* = 281
Attitude toward medication	Mean (SD) DAI-10 score	6.0 (3.66)	5.7 (3.66)	6.1 (3.67)	4.5 (4.03)
Medication satisfaction	Mean (SD) MSQ score	5.8 (1.40)	5.6 (1.49)	5.9 (1.34)	5.2 (1.44)
	“Very satisfied,” % (n/N)	35.0 (200/572)	30.2 (60/199)	37.5 (140/373)	13.8 (37/268)
Quality of life	Mean (SD) QOLI score				
	General life satisfaction	4.8 (1.30)	4.7 (1.39)	4.9 (1.25)	4.3 (1.52)
	Daily activities	4.8 (1.13)	4.7 (1.22)	4.9 (1.07)	4.3 (1.29)
	Family contact	4.9 (1.44)	4.7 (1.56)	5.0 (1.36)	4.5 (1.59)
	Social relations	4.9 (1.20)	4.8 (1.29)	4.9 (1.14)	4.5 (1.22)
	Finance	3.9 (1.50)	3.6 (1.57)	4.0 (1.45)	3.3 (1.71)
	Safety	5.0 (1.26)	5.0 (1.29)	5.0 (1.25)	4.5 (1.58)
	Health	4.6 (1.28)	4.5 (1.39)	4.6 (1.22)	4.0 (1.36)
Patient-caregiver engagement	Mean (SD), frequency of interaction in the past 4 weeks				
	Talk to or email a member of family	3.4 (1.53)	3.6 (1.54)	3.3 (1.52)	3.3 (1.60)
	Get together with family	2.9 (1.49)	2.9 (1.54)	2.8 (1.46)	2.8 (1.50)
	Visit with a friend	2.6 (1.44)	2.4 (1.31)	2.7 (1.50)	2.3 (1.39)
	Talk to or email a friend who does not live with you	2.6 (1.49)	2.6 (1.47)	2.6 (1.50)	2.5 (1.55)
	Plan ahead to do something with another person	2.0 (1.13)	1.9 (1.08)	2.0 (1.15)	1.9 (1.13)
	Spend time with someone more than a friend	2.1 (1.53)	2.1 (1.52)	2.1 (1.53)	2.3 (1.68)
Patient functioning	Mean PSP total score	61.6	59.6	62.7	57.5

*APT* antipsychotic therapy, *DAI-10* Drug Attitude Inventory 10-item scale, *LAI* long-acting injectable (includes paliperidone palmitate long-acting injectable and risperidone long-acting injectable), *MSQ* Medication Satisfaction Questionnaire, *PSP* Personal and Social Performance scale, *QOLI* Lehman’s Quality of Life Interview, *SD* standard deviation

#### Medication satisfaction

**Fig. 4:**
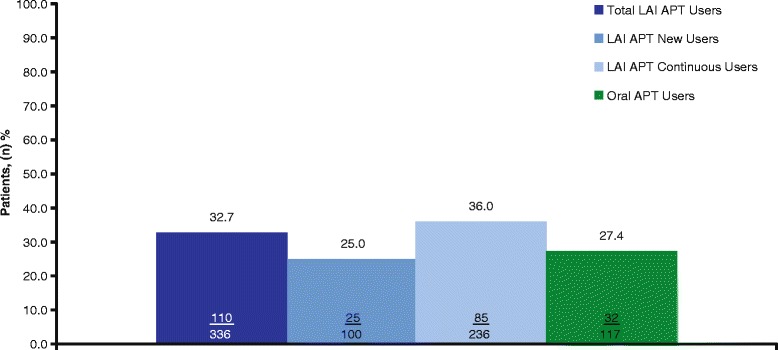
Proportion of patients “very satisfied” with medication at 12-month follow-up.^a^ APT, antipsychotic therapy; LAI, long-acting injectable (includes paliperidone palmitate long-acting injectable and risperidone long-acting injectable). Values in bars are n/N. Total LAI APT = LAI new users + LAI continuous users. ^a^All subjects with schizophrenia

The proportion of patients that reported being “very satisfied” with their current APT was 35% in the LAI APT cohorts and 13.8% in the oral APT cohort at enrollment (Table 4). At the 12-month follow-up visit, patient satisfaction was 27.4% in the oral APT cohort, and the proportion of patients who were “very satisfied” with their current LAI APT ranged from 25.0% to 36.0% (Fig. 4).

#### Quality of life

At the enrollment visit, the mean (SD) score for general life satisfaction was 4.8 (1.30) among patients in the total LAI APT cohorts and 4.3 (1.52) among patients in the oral APT cohort (Table 4). Mean (SD) general life satisfaction scores at the 12-month follow-up visit were 5.0 (1.30) for total LAI APT and 4.8 (1.31) for oral APT cohorts.

Satisfaction at enrollment with respect to daily activities, family contact, social relations, finance, safety, and health are shown in Table 4. Mean (SD) changes in Lehman’s Quality of Life Interview scores at enrollment and at the 12-month follow-up visit are shown in Additional file 3: Figure S1.

#### Alcohol and substance abuse

The mean (SD) number of days that patients used alcohol in the past 30 days was 2.0 (4.92) days for the total LAI APT cohorts at the 12-month follow-up visit. Alcohol use in the past 30 days was 1.9 (4.74) days in the oral APT cohort. At the 12-month follow-up visit, the mean (SD) number of days that patients used >1 substance of abuse per day in the past 30 days was 0.6 (3.19) in the total LAI APT cohort and 2.5 (7.59) in the oral APT cohort. The most commonly used substances of abuse at the 12-month follow-up in the total LAI APT and oral APT cohorts were opiates or analgesics (1.2 [5.08] and 2.7 [8.06] days); sedatives, hypnotics, or tranquilizers (1.8 [6.98] and 2.6 [8.33] days); and cannabis (1.1 [4.00] and 2.1 [6.61] days).

#### Social engagement and patient–caregiver engagement

At enrollment, the extent of patient interaction and engagement with caregivers appeared to be similar between the total LAI APT and oral APT cohorts, except that patients who used LAI APT were more likely to “visit with a friend” than those who used oral APT (Table 4). Mean change from baseline in social engagement and patient–caregiver engagement scores at the 12-month follow-up visit are shown in Additional file 4: Figure S2.

### Clinical assessment*s*

#### Patient functioning

Mean (SD) total PSP scores were 61.6 (15.48) and 57.5 (14.20) in patients in the total LAI APT and oral APT cohorts at the enrollment visit, respectively (Table 4), and 65.2 (16.43) and 61.2 (13.08), at the 12-month follow-up visit, respectively.

#### Remission

At enrollment, the SCI-SR total, positive, and negative scores were all lower in patients in the total LAI APT cohort compared with those in the oral APT cohort, suggesting higher remission in the total LAI APT cohort. A total of 24.4% of patients in the total LAI APT cohort and 9.1% of patients in the oral APT cohort were in remission at enrollment and 40.0% and 23.6% were in remission, respectively, at the 12-month follow-up visit.

### Reasons for initiating medication: Clinician perspective

“Compliance issues” was the most common reason for initiating LAI APT (52.9%). “Insufficient response” was the second most common reason for initiating LAI APT (23.0%).

### Suicidality

Twelve percent of patients in the total LAI APT cohort and 29.5% of patients in the oral APT cohort were suicidal at enrollment. The proportion of patients who were suicidal at enrollment was higher in new users of LAI APT than in continuous users of LAI APT (17.5% vs 9.2%). At the 12-month follow-up visit, the proportion of patients who were suicidal was 9.1% in the total LAI APT cohort and 25.0% in the oral APT cohort. As observed at enrollment, the proportion of patients who were suicidal at the 12-month follow-up visit was higher in new users of LAI APT than in continuous users of LAI APT (13.6% vs 7.6%).

## Discussion

The objective of REACH-OUT was to provide health care providers, researchers, policy makers, and other stakeholders a holistic picture of real-world schizophrenia treatment practices in naturalistic, community settings in the United States. At enrollment, approximately two-thirds of recruited patients were receiving LAI APT and approximately one-third of recruited patients were receiving oral APT. Overall, a number of differences in patient characteristics and outcomes were observed between patients in the LAI APT cohort and the oral APT cohort. It is noteworthy given that patients in the LAI APT cohorts were significantly younger when first diagnosed and first hospitalized compared with those in the oral APT group, suggesting that the LAI APT cohorts had a longer history of disease at enrollment.

In the United States, the use of LAI APT is often reserved for persons with more chronic, long-standing disease and a history of poor efficacy and/or poor adherence to oral APT regimens, despite several recent studies that have demonstrated the efficacy and safety of LAI APT in patients with early illness [40–44]. The acceptability of prescribing LAI APT to patients with first-episode psychosis is currently the subject of debate [45]. However, some European countries use LAI APT in younger, recently diagnosed patients. The reasons underlying regional differences in these prescribing patterns are unknown but may reflect differences in clinical practice or market access to LAI APT.

Real-world, observational studies play an important role in supporting the evidence base for drugs and therapies, prescribing decisions, and patient management [46, 47]. To date, prospective observational studies evaluating the real-world treatment of patients with mental illness have been predominantly conducted internationally with a focus on RLAI [23–28]. REACH-OUT is unique in that it was conducted in outpatient settings exclusively in the United States and encompassed several APT treatments. Data from REACH-OUT add to the body of evidence for schizophrenia management by providing outcomes data in patients treated with atypical LAI APTs (RLAI and PP LAI) and various oral APTs. Our observations generally agree with those of another US-based study: Schizophrenia Outcomes–Utilization Relapse and Clinical Evaluation (SOURCE) [29, 30]. Results of SOURCE—a 24-month, multicenter, prospective, longitudinal, observational study in patients with schizophrenia who were initiated on RLAI—are generally comparable to our REACH-OUT findings in that LAI APT was associated with improvements in daily functioning and a decrease in hospitalizations [29, 30]. It should be noted, however, that outcomes associated with APTs vary according to study design, with observational studies favoring depot over oral medication formulations [48].

Several limitations should be considered when interpreting the findings of this study. First, no intervention was provided, and APT selection was at the discretion of the treating physician. APTs were not randomly assigned, and this could contribute to an imbalance in known and unknown patient characteristics between study cohorts. Second, the types of oral APTs prescribed were not recorded, and all patients prescribed an oral medication were placed in the oral APT group, precluding any analyses based on individual oral APTs. Third, to account for the recent launch of PP LAI in the US market, study enrollment was expanded for PP LAI by including patients who were on continuous PP LAI for any time period prior to enrollment. Fourth, new and continuous user groups were only established in the LAI APT group to enhance enrollment of patients using LAI APT, given the low use of LAI APTs in the US market. All comparisons were made to the oral APT group, who were considered new users by definition. Fifth, the 12-month data include only those patients who completed 12 months of study participation, and improvements measured by mean change from enrollment values might not be generalizable to the entire cohort. Sixth, the true baseline patient characteristics and true baseline values for study outcomes could not be collected for the continuous LAI APT users, who comprised 64.3% of the LAI APT cohort. Consequently, most of the LAI APT users might have reached maintenance treatment at study enrollment, making it challenging to detect further improvement in the outcomes 12 months after enrollment. It should also be noted that the distribution of LAI APT usage (63.5%) versus oral APT usage (29.8%) does not generally represent actual treatment practice or prescription patterns in the United States. Finally, because this study was restricted to patients treated at CBHO sites, the study results may not be generalizable to the entire population of patients with schizophrenia treated at other types of treatment settings (eg, correctional settings, private practices, hospitals, pharmacies). As this is a descriptive analysis, it does not account for the baseline differences that may exist due to the nature of the study design or the use of different antipsychotics used in general clinical practice for schizophrenia.

## Conclusions

The findings from this observational study suggest potential differences in the patient profiles, treatment patterns, and clinical and economic outcomes among patients with schizophrenia treated with LAI APT or oral APT at CBHOs. These results may be useful for generating hypotheses for future studies. Further comparative analysis that adjusts for nonrandom treatment assignment is needed to better evaluate the impact of treatment selection (LAI APT vs oral APT) on these patient outcomes.

## Additional files

Additional file 1: Table S1. Ethics committees by study site. (DOCX 13 kb)

Additional file 2: Table S2. Outpatient utilization at enrollment and at the 12-month follow-up visit. (DOCX 14 kb)

Additional file 3: Figure S1. Mean change from baseline (enrollment) in Lehman’s Quality of Life Interview scores (12-month follow-up visit). APT, antipsychotic therapy; LAI, long-acting injectable (includes paliperidone palmitate long-acting injectable and risperidone long-acting injectable); QOLI, Quality of Life Interview. Total LAI APT = LAI new users + LAI continuous users. (EPS 2025 kb)

Additional file 4: Figure S2. Mean change from baseline in social engagement and patient–caregiver engagement (12-month follow-up visit). APT, antipsychotic therapy; LAI, long-acting injectable (includes paliperidone palmitate long-acting injectable and risperidone long-acting injectable); QOLI, Quality of Life Interview. Total LAI APT = LAI new users + LAI continuous users. (EPS 2044 kb)

## Abbreviations

APT: antipsychotic therapy
ASI-Lite: Addiction Severity Index–Lite
CBHO: community behavioral health organizations
CI: confidence interval
DAI-10: Drug Attitude Inventory 10-item scale
HR: hazard ratio
LAI: long-acting injectable
MSQ: medication satisfaction questionnaire
PP LAI: paliperidone palmitate long-acting injectable
PSP: Personal and Social Performance scale
REACH-OUT: Research and Evaluation of Antipsychotic Treatment in Community Behavioral Health Organizations, Outcomes
RLAI: risperidone long-acting injectable
SCI-SR: Structured Clinical Interview for Symptoms of Remission
SOURCE: Schizophrenia Outcomes–Utilization Relapse and Clinical Evaluation
