# Supplementary material for: The Research and Evaluation of Antipsychotic Treatment in Community Behavioral Health Organizations, Outcomes (REACH-OUT) study: real-world clinical practice in schizophrenia
Source: BMC Psychiatry. 2018 Jan 29;18:24. doi: 10.1186/s12888-018-1594-1 (PMC5789676; doi:10.1186/s12888-018-1594-1)
Supplement: Supplementary file 1 — Ethics committees by study site. (DOCX 13 kb) [file 12888_2018_1594_MOESM1_ESM.docx]

**Additional Table 1** Ethics committees by study site

| Site No. | Site | IRB/EC Name |
| --- | --- | --- |
| 101 | Bergen Regional Medical Center | New England Institutional Review Board |
| 105 | Center for Health Care Services | New England Institutional Review Board |
| 106 | Centerpointe Research Institute | New England Institutional Review Board |
| 107 | Centerstone Research Institute | Centerstone Institutional Review Board |
| 108 | Chestnut Ridge Counseling Services, Inc. | New England Institutional Review Board |
| 109 | Family Services of Western PA | New England Institutional Review Board |
| 110 | Frontier Institute | New England Institutional Review Board |
| 111 | Gateway Healthcare | New England Institutional Review Board |
| 117 | Medical Development Centers LLC (Opelousas) | New England Institutional Review Board |
| 120 | South Shore Mental Health Center | New England Institutional Review Board |
| 121 | Sun Valley Research Center | New England Institutional Review Board |
| 122 | OCCMHA/Training and Treatment Innovations: Oakland County Community Mental Health Authority | New England Institutional Review Board |
| 123 | The Mental Health Center of Greater Manchester | State of New Hampshire DHHS Committee for Protection of Human Subjects |
| 124 | Institute for Health Evaluation and Research Unlimited (IHEARU) | Mental Health Mental Retardation of Tarrant County IRB |
| 126 | Cherry Street Health Services | New England Institutional Review Board |
| 127 | Aurora Mental Health Center | New England Institutional Review Board |
| 132 | River Edge Behavioral Health Center | New England Institutional Review Board |
| 134 | Outpatient Psychiatry Rush University Medical Center | New England Institutional Review Board |
| 135 | Southern Illinois University School of Medicine | Springfield Committee for Research Involving Human Subjects |
| 136 | Community Mental Health Center Inc. | New England Institutional Review Board |
| 137 | Bootheel Counseling Services | New England Institutional Review Board |
| 138 | Burrell Behavioral Health | New England Institutional Review Board |
| 139 | Carolina Behavioral Care Durham (CAREMARK) | New England Institutional Review Board |
| 140 | APF Research | New England Institutional Review Board |
| 141 | St. Joseph’s Regional Medical Center | St. Joseph's Regional Medical Center IRB |
| 142 | Lehigh Valley Hospital Department of Psychiatry | Lehigh Valley Health Network Research Participant Protection Office |
| 143 | Carolina Behavioral Care Henderson | New England Institutional Review Board |
| 144 | Centerstone Research Center | Centerstone Institutional Review Board |
| 145 | Shanti Clinical Trials | New England Institutional Review Board |
| 147 | OCCMHA/Easter Seals Oakland County Community Mental Health Authority | State of Michigan Institutional Review Board |
| 148 | South Coast Clinical Trials, Inc (Norwalk) | New England Institutional Review Board |
| 149 | Carolina Behavioral Care Hillsborough | New England Institutional Review Board |
| 150 | Medical Development Centers LLC (Baton Rouge) | New England Institutional Review Board |
| 151 | LifeStream Behavioral Center, Inc | New England Institutional Review Board |
| 152 | Greater Nashua Mental Health Center | New England Institutional Review Board |
| 153 | Premier Psychiatric Research Institute | New England Institutional Review Board |
| 154 | OCCMHA/Community Network Services, Inc.: Oakland County Community Mental Health Authority | New England Institutional Review Board |
| 156 | Human Services Center (Fayette Companies) | New England Institutional Review Board |
| 158 | Seminole Behavioral Healthcare | New England Institutional Review Board |
| 160 | South Coast Clinical Trials (Anaheim) | New England Institutional Review Board |
| 161 | Alvarado Parkway Institute | New England Institutional Review Board |
| 162 | Neuropsychiatry Clinic and Research Center | New England Institutional Review Board |
| 165 | Unison/Reynolds Clinic | New England Institutional Review Board |
| 166 | Unison/Unison Behavioral Health Group | New England Institutional Review Board |
| 167 | Diligent Clinical Trials | New England Institutional Review Board |
| 168 | Century Health Inc. | New England Institutional Review Board |
| 169 | Santa Cruz Behavioral | New England Institutional Review Board |
