# Supplementary material for: The Research and Evaluation of Antipsychotic Treatment in Community Behavioral Health Organizations, Outcomes (REACH-OUT) study: real-world clinical practice in schizophrenia
Source: BMC Psychiatry. 2018 Jan 29;18:24. doi: 10.1186/s12888-018-1594-1 (PMC5789676; doi:10.1186/s12888-018-1594-1)
Supplement: Supplementary file 2 — Outpatient utilization at enrollment and at the 12-month follow-up visit. (DOCX 14 kb) [file 12888_2018_1594_MOESM2_ESM.docx]

**Additional Table 2.** Outpatient utilization at enrollment and at the 12-month follow-up visit.

| **Outpatient Utilization Measures** | **LAI APT** | | | | | |  | |
| --- | --- | --- | --- | --- | --- | --- | --- | --- |
|  | **Total n = 599** | | **New Users**  **n = 214** | | **Continuous Users n = 385** | | **Oral APT  n = 281** | |
| **Enrollment Visit** | **Mean (SD)** | **Change From BL** | **Mean (SD)** | **Change From BL** | **Mean (SD)** | **Change From BL** | **Mean (SD)** | **Change From BL** |
| Nurse practitioner visits | 1.3 (2.52) | **⎯** | 0.8 (1.66) | **⎯** | 1.6 (2.84) | **⎯** | 0.7 (1.78) | **⎯** |
| Therapist/counselor visits | 6.5 (20.53) | **⎯** | 2.8 (10.29) | **⎯** | 8.6 (24.10) | **⎯** | 3.3 (12.24) | **⎯** |
| Nurse visits | 6.6 (8.71) | **⎯** | 4.2 (5.27 | **⎯** | 7.9 (9.85) | **⎯** | 2.3 (6.26) | **⎯** |
| Group sessions (with any of the professionals) | 9.7 (29.02) | **⎯** | 7.6 (20.30) | **⎯** | 10.9 (32.77) | **⎯** | 1.3 (7.40) | **⎯** |
| Accessed services at the site, days | 21.9 (38.59) | **⎯** | 15.7 (28.27) | **⎯** | 25.0 (42.51) | **⎯** | 9.7 (16.55) | **⎯** |
| Nights slept at a residential facility that is part of the site | 10.7 (40.38) | **⎯** | 4.8 (25.79) | **⎯** | 13.9 (46.18) | **⎯** | 3.9 (20.93) | **⎯** |
| Canceled one-on-one visits at the site | 1.0 (2.31) | **⎯** | 1.1 (2.61) | **⎯** | 1.0 (2.13) | **⎯** | 0.5 (1.25) | **⎯** |
| **12-Month Visit** |  |  |  |  |  |  |  |  |
| Nurse practitioner visits | 0.8 (1.74) | –0.5 | 0.5 (1.36) | –0.3 | 1.0 (1.87) | –0.6 | 0.4 (1.29) | –0.2 |
| Therapist/counselor visits | 5.8 (15.25) | –0.5 | 5.2 (13.89) | 2.1 | 6.1 (15.90) | –1.8 | 2.7 (7.87) | –0.6 |
| Nurse visits | 6.7 (7.29) | 0.3 | 5.9 (5.05) | 1.3 | 7.1 (8.14) | –0.2 | 1.6 (3.11) | –0.3 |
| Group sessions (with any of the professionals) | 7.2 (22.30) | –1.4 | 10.7 (31.44) | 3.4 | 5.5 (15.88) | –3.8 | 1.9 (8.47) | 0.9 |
| Accessed services at the site, days | 24.7 (42.99) | 2.2 | 18.8 (34.37) | 1.0 | 27.5 (46.32) | 2.8 | 8.6 (17.51) | –1.9 |
| Nights slept at a residential facility that is part of the site | 11.7 (43.35) | –0.7 | 7.1 (33.38) | 0.3 | 13.9 (47.30) | –1.3 | 2.0 (16.50) | 0.6 |
| Canceled one-on-one visits at the site | 0.9 (2.13) | –0.1 | 0.7 (1.58) | –0.4 | 1.0 (2.35) | 0.1 | 1.1 (2.38) | 0.5 |

APT, antipsychotic therapy; BL, baseline; LAI, long-acting injectable (includes paliperidone palmitate long-acting injectable and risperidone long-acting injectable); SD, standard deviation.
